# Inter and intra-rater repeatability of the scoring of foot pain drawings

**DOI:** 10.1186/1757-1146-6-44

**Published:** 2013-11-01

**Authors:** Benjamin D Chatterton, Sara Muller, Martin J Thomas, Hylton B Menz, Keith Rome, Edward Roddy

**Affiliations:** 1 Arthritis Research UK Primary Care Centre, Research Institute for Primary Care and Health Sciences, Keele University, Staffordshire ST5 5BG, United Kingdom; 2 Lower Extremity and Gait Studies Program, Faculty of Health Sciences, La Trobe University, Bundoora, VIC 3086, Australia; 3 Health and Rehabilitation Research Institute and School of Podiatry, AUT University, Auckland 0627, New Zealand

**Keywords:** Foot pain, Pain drawings, Manikins, Reliability, Repeatability, Agreement

## Abstract

**Background:**

Foot pain drawings (manikins) are commonly used to describe foot pain location in self-report health surveys. Respondents shade the manikin where they experience pain. The manikin is then scored via a transparent overlay that divides the drawings into areas. In large population based studies they are often scored by multiple raters. A difference in how different raters score manikins (inter-rater repeatability), or in how an individual rater scores manikins over time (intra-rater repeatability) can therefore affect data quality. This study aimed to assess inter- and intra-rater repeatability of scoring of the foot manikin.

**Methods:**

A random sample was generated of 50 respondents to a large population based survey of adults aged 50 years and older who experienced foot pain and completed a foot manikin. Manikins were initially scored by any one of six administrative staff (Rating 1). These manikins were re-scored by a second rater (Rating 2). The second rater then re-scored the manikins one week later (Rating 3). The following scores were compared: Rating 1 versus Rating 2 (inter-rater repeatability), and Rating 2 versus Rating 3 (intra-rater repeatability). A novel set of clinically relevant foot pain regions made up of one or more individual areas on the foot manikin were developed, and assessed for inter- and intra-rater repeatability.

**Results:**

Scoring agreement of 100% (all 50 manikins) was seen in 69% (40 out of 58) of individual areas for inter-rater scoring (range 94 to 100%), and 81% (47 out of 58) of areas for intra-rater scoring (range 96 to 100%). All areas had a kappa value of ≥0.70 for inter- and intra-rater scoring. Scoring agreement of 100% was seen in 50% (10 out of 20) of pain regions for inter-rater scoring (range 96 to 100%), and 95% (19 out of 20) of regions for intra-rater scoring (range 98 to 100%). All regions had a kappa value of >0.70 for inter- and intra-rater scoring*.*

**Conclusions:**

Individual and multiple raters can reliably score the foot pain manikin. In addition, our proposed regions may be used to reliably classify different patterns of foot pain using the foot manikin.

## Background

Foot pain is a common occurrence in the general adult population, with an estimated prevalence of between 17 and 24% [1,2]. The prevalence increases with age [2], and in older people foot pain is associated with increased risk of falls [3], locomotor disability [4], impairment of activities of daily living [2,5,6], and significantly reduced health-related quality of life [1]. Eight per cent of musculoskeletal consultations in primary care are related to foot and ankle problems [7].

The accurate assessment of foot pain is therefore important in both clinical practice and epidemiological research. However, there is a large variation in the diagnosis of foot problems by primary care physicians [8], and many junior doctors do not feel confident in the assessment of the foot [9]. In the research setting, a reproducible way of localising foot pain is required, as patients may have difficulty in accurately describing their foot problems [10]. Any self-report questionnaires used for this purpose must also account for the literacy level of the general population, and accommodate those who respond to a visual rather than verbal form of questioning [10].

Pain drawings (also known as manikins) are a useful tool to address these issues and assess pain location in these contexts. A manikin of the whole body or a body part is provided, and respondents are asked to shade on the manikin any area where they experience pain. A transparent overlay divided into mutually exclusive areas is placed over the completed drawing, allowing pain location to be categorised [11]. Combinations of these areas can also be grouped together to classify different pain regions, for example a specific body region made up of several mutually exclusive areas, or to distinguish widespread pain from localised pain [11].

**Figure 1:**
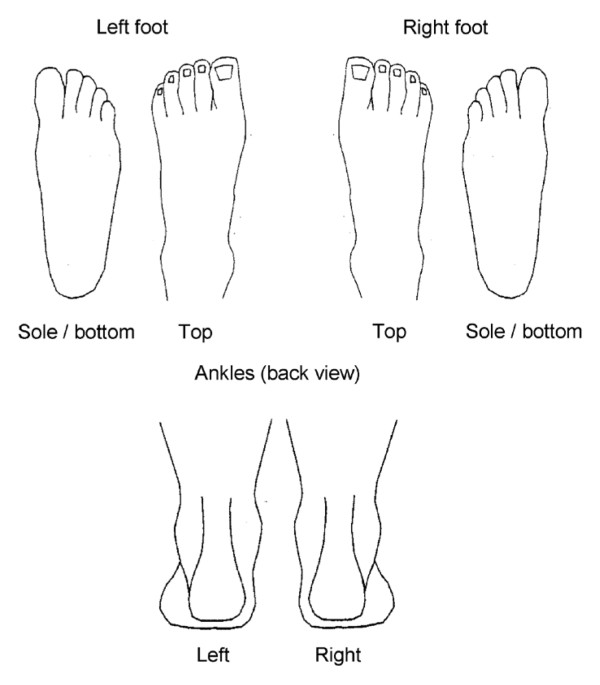
**The blank foot pain manikin.** Garrow AP, Silman AJ, Macfarlane GJ: The Cheshire Foot Pain and Disability Survey: a population survey assessing prevalence and associations. *Pain* 2004, 110:378-84. This figure has been reproduced with permission of the International Association for the Study of Pain**®** (IASP). The figure may NOT be reproduced for any other purpose without permission.

The foot pain manikin (© The University of Manchester 2000. All rights reserved), developed by Garrow *et al.*[2], is a manikin specific to the foot and ankle that includes six drawings; the dorsal, plantar and posterior aspects of each foot (Figure 1). It has previously been used in epidemiological studies [2,12], and similar foot and ankle manikins have been proposed as a screening tool to identify foot and ankle problems in the clinical setting [10].

Good test-retest reliability has previously been reported for respondent-completed manikins [13]. However, a potential disadvantage of these manikins is that completed pain drawings are often scored by multiple administrative staff, particularly in large epidemiological studies. Therefore, a difference in how raters interpret the shading on the completed manikins (the inter-rater repeatability) is a potential source of reduced data quality. Similarly, if there is a lack of consistency in how an individual rater scores the manikins (the intra-rater repeatability), then this could also affect data quality [11].

There are few studies on inter- and intra-rater repeatability of pain drawings. Lacey *et al.* reported complete scoring agreement between eight different raters in 49 of 50 whole body pain manikins used to assess the presence of widespread pain [11]. More recently Persson *et al.* reported good inter- and intra-rater reliability in electronically scored pain drawings, where completed whole body manikins were scanned into a specialised computer programme, and any shaded areas encircled digitally with a computer mouse [14]. However, to date there have been no studies that assess the repeatability of the foot pain manikin. Therefore, this study aimed to assess the repeatability of the scoring of the foot manikin, assessing this at both the inter- and intra-rater levels.

## Methods

### Study design

Ethical approval was obtained from the Coventry Research Ethics Committee (10/H1210/5). All adults aged 50 years and over registered at four general practices in North Staffordshire, United Kingdom were sent a postal Health Survey questionnaire as part of the Clinical Assessment Study of the Foot (CASF) [12]. The questionnaire contained the filter question “In the past month, have you had any ache or pain that has lasted for one day or longer in your feet?”, with two tick boxes corresponding to yes or no. If the respondent ticked “Yes”, they were directed to the following instructions: “Please shade in the diagrams below any pain you have had in your feet in the last month that has lasted one day or longer.” Below this statement was the foot pain manikin proposed by Garrow *et al.*, showing the dorsal, plantar and posterior aspects of each foot (© The University of Manchester 2000. All rights reserved) [2].

### Manikin scoring technique

**Figure 2:**
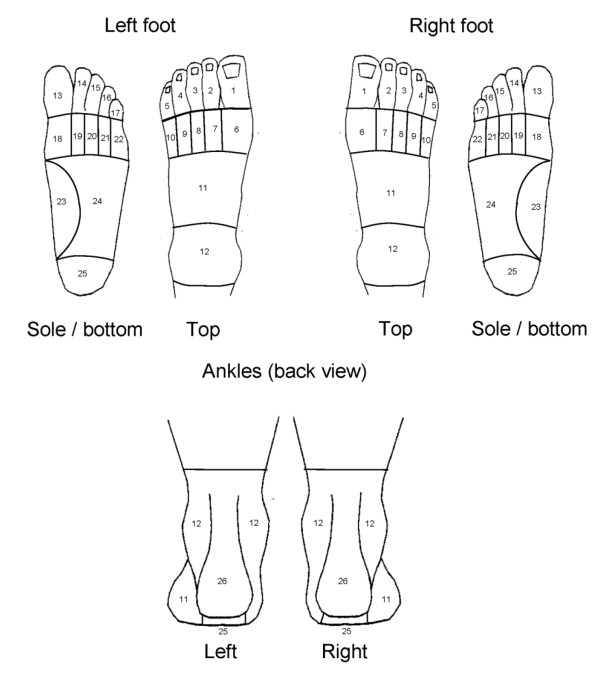
The areas used for scoring of the foot pain manikin.

The foot pain manikin was scored using a transparent overlay dividing the foot images into 26 mutually exclusive areas (Figure 2), as previously described by Garrow *et al.*[2]. The scoring was entered into a database, coded as a “1” if an area was shaded and a “0” if it was not shaded. The guidance given to raters for scoring the manikin were as follows: (i) If any part of the mark (e.g. scribble, shading, cross) no matter how small or faint is within a template area then code it as 1 (for shading) in the database; (ii) If a mark (e.g. scribble, shading, cross) goes over two (or more) template areas then code them both (all); (iii) If an arrow is touching a coded area, score on the database as 1; (iv) Any shading outside the template is not to be coded. Returned questionnaires were scored and coded by one of six, non-clinical administrative staff. These staff had no prior experience in scoring pain manikins, and were trained by an administrator with previous experience in manikin scoring, in addition to receiving the above instructions.

### Assessment of inter- and intra-rater agreement in scoring of individual areas

To assess the inter- and intra-rater agreement of scoring, a random sample of 50 previously scored and coded questionnaires was selected in which respondents had answered yes to the initial foot pain filter question (Rating 1). This size of sample was chosen as it has previously been suggested to be sufficient for repeatability studies using the kappa statistic [15]. This sample size has also been used in previous studies assessing repeatability of whole body pain drawings [11]. To assess the inter-rater agreement, the foot manikins from the random sample were re-scored by a second rater (BDC) (Rating 2), blind to the original scoring, and compared with the scoring of the initial raters from the CASF. To assess intra-rater agreement, the second rater (BDC) re-scored the random sample a week after scoring them initially (Rating 3). These scores were then compared: Rating 1 versus Rating 2, and Rating 2 versus Rating 3. The second rater (BDC) had no prior experience in scoring pain drawings, and was issued with the same instructions as given to the raters from rating 1 listed above. No formal training was given.

### Categorisation of foot pain areas and assessment of inter- and intra-rater agreement

In an effort to aid classification of different types of foot pain, 10 regions comprising one or more clinically-relevant pain areas were developed. The choice of foot pain regions and how they would be defined were agreed by consensus discussion between HBM, KR, MJT and ER. The agreed foot pain regions were: the first metatarsophalangeal (1^st^ MTP) joint, hallux, great toe, lesser toes, plantar forefoot, midfoot, medial arch, ankle, plantar heel and posterior heel (Table 1). Individual areas of the foot manikin were combined so as to give a score of 1 for a foot region if one or more of the individual areas within the region was shaded and as a 0 for the absence of shading in all areas. Inter- and intra-rater agreement was compared in the same way as for the scoring of individual areas, using the same random sample of 50 manikins from the CASF.

**Table 1:** Categorisation of foot manikin pain regions

**Region term**	**Manikin view (s)**	**Numbered areas (see Figure**2**)**
First MTPJ	Foot only	6 or 18
Hallux	Foot only	1 or 13
Great toe	Foot only	1, 6, 13 or 18
Lesser toes	Foot only	2, 3, 4, 5, 7, 8, 9, 10, 14, 15, 16, 17, 19, 20, 21, or 22
Plantar forefoot	Foot only	18, 19, 20, 21, or 22
Midfoot	Foot only	11, 23 or 24 (excludes area 11 on ankle view)
Medial arch	Foot only	23
Ankle	Foot or ankle	12
Plantar heel	Foot or ankle	25
Posterior heel	Ankle only	26

### Overall reliability

As a crude measure of overall non-area specific, inter- and intra-rater reliability, the total number of areas scored as containing shading for each respondent at different scorings of the data were compared [11].

### Statistical analysis

The prevalence of pain in each area of the foot manikin and each defined pain region was calculated as the median of the prevalence calculated in each of the two ratings being compared. Inter- and intra-rater agreement for both individual areas scored on the foot manikin and the newly-proposed pain regions were assessed via two methods. First, the percentage of drawings for which there was complete agreement in scoring of an area or region between raters was calculated (the absolute percentage of agreement). Second, Cohen’s Kappa coefficient (κ) and the associated lower limit of the 95% 1-sided confidence interval were calculated for the different ratings to allow adjustment for agreement attributable to chance. A positive rating for agreement was defined as κ ≥ 0.70, as suggested by Terwee *et al.*[15]. For analysis of individual areas and foot regions, the analysis was conducted separately for each foot. Intraclass correlation coefficients for agreement (ICC_agreement_[1,2], 2-way random effects model) and associated 95% confidence intervals were calculated from the total number of areas positively scored at different scorings to assess overall reliability [16]. Coding of the manikin scoring was performed with Microsoft Access 2010, and data analysis was performed with SPSS Statistics for Windows (version 20.0, IBM Corp., Armonk, NY, 2011).

## Results

### Demographics

Fifty respondents from the CASF study were randomly selected, of whom 26 were female (52%). The mean age of male respondents was 66.3 ± 8.3 years, and the mean age of female respondents was 64.8 ± 9.8 years.

### Inter and intra-rater agreement in scoring of individual areas

The median prevalence of positive scoring for each individual pain area is shown in Additional file 1. For inter-rater scoring, the most commonly shaded area(s) were areas 11 and 12 of the left foot (27%), area 11 of the right foot (26%), area 26 of the left ankle (26%), and 12 and 26 (20%) of the right ankle. For intra-rater scoring, the most commonly shaded areas were area 12 of the left foot (40%), area 11 of the right foot (32%), area 12 of the left ankle (39%), and 12 and 26 (32%) of the right ankle.

Scoring agreement of 100% (all 50 manikins) was seen in 69% (40/58) of areas for inter-rater scoring, and 81% (47/58) of areas for intra-rater scoring. Agreement ranged from 94% (47/50 manikins) to 100% in the inter-rater analysis, and from 96% (48/50 manikins) to 100% in the intra-rater analysis. The κ values ranged from 0.70 to 1.00 in the inter-rater analysis, and 0.81 to 1.00 in the intra-rater analysis (Additional file 1). The area with the least agreement for both inter and intra-rater scorings was area 4 (the fourth toe) of the right foot, with agreement of 94% (κ = 0.70) for inter-rater scoring, and agreement of 96% (κ = 0.81) for intra-rater scoring.

### Inter and intra-rater agreement in scoring of foot pain regions

The median prevalence of positive scoring for the foot pain regions defined in Table 1 is seen in Table 2. For inter-rater scoring, the most commonly shaded regions for both feet were the left midfoot (44%) and the right midfoot and ankle (34%). For intra-rater scoring, the most commonly shaded regions for both feet were the left ankle (53%) and the right midfoot and right ankle (45%).

**Table 2:** Inter- and intra-rater reliability of foot pain regions

	**Inter-rater**			**Intra-rater**		
Region*	**Median% prevalence of pain**	**No. of pain drawings agreed on (%)**	**Κ (95% confidence limit)**^**†**^	**Median% prevalence of pain**	**No. of pain drawings agreed on (%)**	**Κ (95% confidence interval)**^**†**^
**L 1st MTPJ**	40	49 (98)	0.96 (0.88)	45	50 (100)	1.00 (1)
**R 1st MTPJ**	24	49 (98)	0.95 (0.86)	31	50 (100)	1.00 (1)
**L Hallux**	21	50 (100)	1.00 (1)	22	50 (100)	1.00 (1)
**R Hallux**	22	50 (100)	1.00 (1)	26	50 (100)	1.00 (1)
**L Great toe**	40	49 (98)	0.96 (0.88)	45	50 (100)	1.00 (1)
**R Great toe**	28	49 (98)	0.96 (0.87)	37	50 (100)	1.00 (1)
**L Lesser toes**	37	50 (100)	1.00 (1)	45	50 (100)	1.00 (1)
**R Lesser toes**	30	49 (98)	0.96 (0.88)	39	50 (100)	1.00 (1)
**L Plantar forefoot**	24	50 (100)	1.00 (1)	26	50 (100)	1.00 (1)
**R Plantar forefoot**	20	50 (100)	1.00 (1)	22	50 (100)	1.00 (1)
**L Midfoot**	44	50 (100)	1.00 (1)	53	50 (100)	1.00 (1)
**R Midfoot**	34	48 (96)	0.92 (0.81)	45	50 (100)	1.00 (1)
**L Medial arch**	15	49 (98)	0.94 (0.82)	21	50 (100)	1.00 (1)
**R Medial arch**	12	50 (100)	1.00 (1)	16	50 (100)	1.00 (1)
**L Ankle**	38	48 (96)	0.92 (0.81)	53	50 (100)	1.00 (1)
**R Ankle**	34	50 (100)	1.00 (1)	45	50 (100)	1.00 (1)
**L Plantar heel**	21	50 (100)	1.00 (1)	24	49 (98)	0.95 (0.84)
**R Plantar heel**	13	50 (100)	1.00 (1)	16	50 (100)	1.00 (1)
**L Posterior heel**	26	49 (98)	0.96 (0.87)	32	50 (100)	1.00 (1)
**R Posterior heel**	20	49 (98)	0.95 (0.86)	29	50 (100)	1.00 (1)

* L = left, R = right.

† Lower limit of the 95% 1-sided confidence interval.

Scoring agreement of 100% (all 50 manikins) was seen in 50% (10/20) of regions for inter-rater scoring, and 95% (19/20) of regions for intra-rater scoring. Agreement ranged from 96% (48/50 manikins) to 100% for inter-rater scoring, and from 98% (49/50 manikins) to 100% for intra-rater scoring. The κ values ranged from 0.92 to 1.00 in the inter-rater scoring, and 0.95 to 1.00 in the intra-rater scoring (Table 2). The regions of least agreement for inter-rater scoring were the left ankle and right midfoot (96% agreement, κ = 0.92), and the left plantar heel for the intra-rater scoring (98% agreement, κ = 0.95).

### Overall non-area specific inter and intra-rater reliability

The mean number of positively coded pain areas for the different ratings was; 9.86 (Rating 1), 10.32 (Rating 2), and 10.12 (Rating 3). The overall inter-rater reliability for the number of positive pain areas recorded was ICC_agreement_ (2, 1) = 0.996 (95% CI 0.990-0.998). The overall intra-rater reliability for the number of positive pain recorded areas was ICC_agreement_ (2, 1) = 0.999 (95% CI 0.997-0.999).

## Discussion

The results of this study show excellent agreement for both inter- and intra-rater scoring of individual areas on the foot pain manikin, with all areas showing a κ value of ≥0.70. In addition, the newly proposed pain regions were found to have high agreement; all regions showed a κ value of >0.70 for both inter- and intra-rater scoring. Non-area specific overall reliability was also excellent, with ICC_agreement_ (2, 1) = 0.99 for both inter- and intra-rater scoring.

Pain drawings are frequently used to assess self-reported pain in both clinical practice and population-based research [1,2,10,12]. Multiple administrative staff are likely to score pain drawings in large population-based surveys. Differences in how different raters interpret completed drawings, and how an individual interprets them over time may therefore reduce data quality. Previous studies have shown good inter- and intra-rater agreement in scoring of pain drawings [11,17-19], although the majority of these were using whole body manikins in the clinical setting.

This is the first study to assess inter- and intra-rater repeatability of the scoring of foot pain drawings currently in use in epidemiological research [2,12]. Our results show that individual and multiple raters can reliably score the foot pain manikin. In addition, the newly developed pain regions may be used to reliably classify foot pain location. These new regions can therefore be used in further epidemiological research using the foot manikin. Although the foot manikin is unable to identify the underlying pathology causing foot pain, reliable pain region classifications may provide an insight into region specific pathologies affecting the foot in population-based studies. Similarly, these new regions could potentially be used as a screening tool for different foot-region specific pathologies in the clinical setting [10].

A limitation of this study is that it is unknown whether shading on the Garrow foot manikin [2] gives an accurate interpretation of the actual anatomical location of a respondent’s foot pain. Waller *et al.*[10] reported the use of a different foot manikin showing the dorsal, plantar and medial aspects of both feet as part of the Swindon Foot and Ankle Questionnaire. Patients who completed the drawings were clinically assessed, and the clinical findings compared to the drawing, with 71% of patients felt to have completed the drawings accurately. Other types of pain drawing have been shown to correlate well with clinical findings. For example, pain drawings used in the assessment of lower back pain have been shown to accurately predict the presence of intervertebral disc pathology [20] and the level of lumbar disc disruption [21], as confirmed by computed tomography/discography.

A further limitation of this study is that we have not assessed the respondent test-retest reliability of the foot pain manikin. Previous studies have reported good test-retest reliability for pain drawings used in the assessment of knee pain [13], lower back pain [22,23], and whole body pain [24], but this is as yet unassessed for the foot pain manikin. It is also worth noting that the random sample from Rating 1 of the pain drawings in the CASF study represented multiple different raters, rather than one individual. Therefore, when assessing the inter-rater agreement, the actual comparison was between an individual (Rating 2, BDC) and a number of different raters. It was reassuring to note that, despite this, good agreement in scoring was observed.

Future studies could further explore the reproducibility and validity of the foot pain manikin and newly identified pain regions. Although we have shown good repeatability of scoring for both individual areas and pain regions on the manikin, the repeatability of shading of these areas and regions by respondents should also be assessed. In addition, to further validate the manikin and pain regions, shading on the manikin could be compared to clinical examination findings and diagnosis.

## Conclusion

The foot manikin can be reproducibly scored by either a single or multiple raters, and it is therefore appropriate for the manikin to be scored by multiple raters in large population based surveys. In addition, we have presented a reproducible set of foot pain categories that may be used to classify foot pain regions in further research that utilises the foot manikin.

## Competing interests

HBM is Editor-in-Chief of the *Journal of Foot and Ankle Research*. It is journal policy that editors are removed from the editorial decision making processes for papers they have co-authored. The remaining authors declare that they have no competing interests.

## Authors’ contributions

ER and SM conceived and designed the study. ER and MJT were responsible for CASF data collection. ER, MJT, KR and HBM created the novel foot pain regions. BDC scored the pain drawings. BDC and SM performed the data analysis. BDC drafted the initial manuscript. All authors read and approved the final manuscript.

## Supplementary Material

Additional file 1 **Inter- and intra-rater reliability of the foot pain manikin by individual area.** This additional table provides the full results for inter- and intra-rater reliability of the foot pain manikin by individual area. The median prevalence of pain, number of pain drawings agreed upon, and Κ statistic are all given for individual areas of the manikin. Click here for file
